# The Human Gastric Microbiome Is Predicated upon Infection with *Helicobacter pylori*

**DOI:** 10.3389/fmicb.2017.02508

**Published:** 2017-12-14

**Authors:** Ingeborg Klymiuk, Ceren Bilgilier, Alexander Stadlmann, Jakob Thannesberger, Marie-Theres Kastner, Christoph Högenauer, Andreas Püspök, Susanne Biowski-Frotz, Christiane Schrutka-Kölbl, Gerhard G. Thallinger, Christoph Steininger

**Affiliations:** ^1^Center for Medical Research, Medical University of Graz, Graz, Austria; ^2^Division of Infectious Diseases, Department of Medicine I, Medical University of Vienna, Vienna, Austria; ^3^Division of Gastroenterology and Hepatology, Department of Internal Medicine, Medical University of Graz, Graz, Austria; ^4^Department of Internal Medicine II, St. John's Hospital Eisenstadt, Eisenstadt, Austria; ^5^Division of Gastroenterology and Hepatology, Department of Internal Medicine III, Medical University Vienna, Vienna, Austria; ^6^Institute of Computational Biotechnology, Graz University of Technology, Graz, Austria; ^7^BioTechMed OMICS Center Graz, Graz, Austria

**Keywords:** *Helicobacter pylori*, CagA, gastric microbiota, multicenter study, 16S rRNA gene analysis

## Abstract

The human gastric lumen is one of the most hostile environments of the human body suspected to be sterile until the discovery of *Helicobacter pylori* (H.p.). State of the art next generation sequencing technologies multiply the knowledge on H.p. functional genomics as well as on the colonization of supposed sterile human environments like the gastric habitat. Here we studied in a prospective, multicenter, clinical trial the 16S rRNA gene amplicon based bacterial microbiome in a total of 30 homogenized and frozen gastric biopsy samples from eight geographic locations. The evaluation of the samples for H.p. infection status was done by histopathology and a specific PCR assay. CagA status was determined by a CagA-specific PCR assay. Patients were grouped accordingly as H.p.-negative, H.p.-positive but CagA-negative and H.p.-positive and CagA-positive (*n* = 10, respectively). Here we show that H.p. infection of the gastric habitat dominates the gastric microbiota in most patients and is associated with a significant decrease of the microbial alpha diversity from H.p. negative to H.p. positive with CagA as a considerable factor. The genera *Actinomyces, Granulicatella, Veillonella, Fusobacterium, Neisseria, Helicobacter, Streptococcus*, and *Prevotella* are significantly different between the H.p.-positive and H.p.-negative sample groups. Differences in microbiota found between CagA-positive and CagA-negative patients were not statistically significant and need to be re-evaluated in larger sample cohorts. In conclusion, H.p. infection dominates the gastric microbiome in a multicentre cohort of patients with varying diagnoses.

## Introduction

The microbiome (the entity of bacteria, viruses, archaea, and fungi) of the human lower intestinal tract has been well characterized in a plethora of clinical and physiological studies (Levy et al., 2016; Tilg et al., 2016; Wehkamp and Frick, 2016) and its significance for medical research, diagnosis, and diseases is increasingly recognized. Correlations between specific patterns of the intestinal microbiome with specific disease entities such as chronic inflammatory bowel disease are undisputed nowadays (Patel et al., 2016; Wehkamp and Frick, 2016). Even microenvironments that were perceived to be sterile in healthy humans such as the urinary bladder or the human lung were found to harbor a diversity of microbes (Cui et al., 2014; Thomas-White et al., 2016). Information on the human gastric microbiome is increasing but still limited and large cohort data are often based on culture dependent approaches or animal models (Khosravi et al., 2014; Majlessi et al., 2017). The human stomach has been supposed to be devoid of significant microbial colonization and diversity because of its hostile environment. The very low pH and tight immune surveillance render the human stomach a gate keeper for the entrance of microbial pathogens into the intestinal tract.

However, the discovery of *Helicobacter pylori* (H.p.) in 1984 (Marshall and Warren, 1984) changed the view on microbial colonization of the stomach. H.p. is one of the genetically best characterized and fully sequenced organisms due to its potentially carcinogenic effect (Wroblewski and Peek, 2016) and high relevance for human health (Tomb et al., 1997; Noto and Peek, 2017; Shah, 2017). Nowadays, multiple other microbiota than H.p. have been described in gastric samples (Bik et al., 2006; Dong et al., 2016; Péré-Védrenne et al., 2017). *Firmicutes, Proteobacteria, Bacteroidetes, Actinobacteria*, and *Fusobacteria* are the most abundant phyla in previous studies detected by culture dependent, mass spectrometry and sequencing approaches (Khosravi et al., 2014; Ianiro et al., 2015; Dias-Jacome et al., 2016). Although, the impact of H.p. on the non-H.p. microbiome has been studied with various techniques in animal models (Kienesberger et al., 2016) as well as human approaches (Bik et al., 2006; Schulz et al., 2016a; Yang et al., 2016; Brawner et al., 2017) the generation of human gastric microbiome and H.p. related data is still of incredible significance to investigate mechanisms of human H.p. infections.

The genome of H.p. is well annotated and pathogenicity islands (PAI) have been described (Feliciano et al., 2015). One of the most virulent of these PAI genes suspected to be a main driver of carcinogenesis is the CagA gene located in the cag PAI (Paredes-Osses et al., 2017). The cag PAI is responsible for translocation of the Cag protein into the host cell (Hatakeyama, 2014). Characterization of the CagA effect in H.p. positive samples on the non-H.p. microbiome has been performed previously in a small Colombian sample cohort. They revealed no statistically significant differences in CagA negative compared to positive infections but showed a trend of reduced H.p. abundance and reduced histopathology score in the CagA negative patients (Yang et al., 2016). Functional studies on the interaction of H.p. and the suspected gastric microbiome were described from *in vitro* studies or mouse models (Khosravi et al., 2016; Kienesberger et al., 2016).

Here we provide NGS based 16S rRNA gene data on the relevance of CagA positive and CagA negative H.p. infections on the gastric microbiome of a clinically very well characterized, adult human population collected at eight different geographic locations all over Austria.

## Materials and methods

### Clinical samples

Two gastric mucosal biopsy samples from the antrum of the stomach were collected from 30 patients, who were older than 18 years and did not have a gastroscopic investigation in the past 10 years. All samples were taken from the antrum region to overcome the problem of variation potentially caused by different gastric regions. The patients included in this study have never been treated for H.p. infection before and were not treated with proton-pump inhibitors for at least 2 weeks or antibiotics for at least 1 month before endoscopy. The indication for gastroscopy was in the majority of the patients upper abdominal pain and suspected gastritis (33%) and symptoms compatible with reflux esophagitis (23%). In this prospective study, samples were grouped according to their H.p. and CagA status but not according to their diagnosis. Samples were selected randomly from a cohort of more than 2000 human gastric biopsies (Bilgilier et al., 2017). A written informed consent was obtained from all participants. The study protocol was approved by the ethics committee of the Medical University Vienna (EK# 1548/2014) and the study was conducted in accordance with the Declaration of Helsinki.

### Evaluation of H. p. infection status in gastric biopsies

Histomorphological evaluation of one of the two gastric biopsy samples for the presence of Helicobacter-like organisms (HLO) was done with the use of hematoxylin and eosin staining and a modified Giemsa staining followed by microscopic evaluation according to standard procedures (Fallone et al., 1997) and as described in detail in Bilgilier et al. (2017). For the detection of H.p.-specific DNA, the second fresh gastric biopsy sample from each patient was homogenized in 1 ml 0.9% NaCl solution (Sigma Aldrich) using Lysing Matrix D tubes with ceramic beads (MP Biomedicals) in an FastPrep®-24 Instrument (MP Biomedicals) at 4.5m/s for 20 s once. An aliquot of 50 μl from the homogenates were subjected to genomic DNA isolation with the QIAamp DNA Mini Kit (Qiagen, Hilden, Germany) immediately according to manufacturer's instructions. The remaining 950 μl homogenate from each sample was frozen without DNA isolation and stored at −80°C until DNA isolation for microbiome analysis. Subsequently, 2 μl of total DNA were used in an H.p. specific PCR assay detecting the 23S rRNA gene by fluorescence detection as described previously (Schabereiter-Gurtner et al., 2004). In brief, this PCR assay was performed with 40 amplification cycles of denaturation at 95°C for 5 s, annealing at 65°C for 10 s and extension at 72°C for 6 s, and read-outs were based on single fluorescence acquisition at the end of each extension step. The presence of CagA in HP positive samples was investigated with use of a PCR assay described previously (Bilgilier et al., 2016). In brief, the combination of three different reverse primers, CagA-rvP1, CagA-rvP2, and CagA-rvP3, allowed the detection of the CagA gene and also typing of the EPIYA motif encoded within this gene, which was considered an important virulence determinant. According to the results from the H.p. specific and the CagA specific PCR assays, the gastric biopsy samples were gathered in three different groups: (Levy et al., 2016) HP negative samples, (Tilg et al., 2016) HP positive samples with HP strains that do not encode the CagA gene and (Wehkamp and Frick, 2016) HP positive samples with HP strains encoding the CagA gene. Each group consisted of 10 individuals.

### DNA isolation for microbiome analysis, 16S rRNA gene PCR amplification and sequencing

Total DNA was isolated by a combination of mechanic and enzymatic lysis according to standard procedures as published recently in Klymiuk et al. (2016). Briefly, fresh biopsy samples were homogenized in 1 ml 0.9% NaCl and 950 μl of the homogenate were frozen and stored at −80°C till DNA isolation. Samples were centrifuged at 6,000 g for 10 min and supernatant was removed. Samples were homogenized in a total volume of 500 μl MagNA Pure Bacteria Lysis Buffer from the MagNA Pure LC DNA Isolation Kit III (Bacteria, Fungi) (Roche, Mannheim, Germany) in MagNA Lyser green beads tubes (Roche, Mannheim, Germany) at 6,500 rpm for 30s repeated three times in a MagNA Lyser Instrument (Roche, Mannheim, Germany). Twenty-five Microliter lysozyme (100 mg/ml) were added to the homogenized samples, mixed and incubated at 37°C for 30 min. Afterwards 43.4 μl Proteinase K (20 mg/ml) were added and samples were incubated at 65°C over night. The next day after heat inactivation of enzymes at 95°C for 10 min DNA was extracted from 250 μl lysed supernatant on a MagNA Pure LC 2.0 (Roche, Mannheim, Germany) according to manufacturer's instructions of the MagNA Pure LC DNA Isolation Kit III (Bacteria, Fungi) (Roche, Mannheim, Germany). Five microliter of total DNA were used in a 25 μl PCR reaction in triplicates using a FastStart High Fidelity PCR system (Roche, Mannheim, Germany). Each PCR reaction comprised of 1x Fast Start High Fidelity Buffer (Roche, Mannheim, Germany), 1.25 U High Fidelity Enzyme (Roche, Mannheim, Germany), 200 μM dNTPs (Roche, Mannheim, Germany), 0.4 μM primers and PCR-grade water (Roche, Mannheim, Germany). For the amplification of phylogenetic informative hypervariable regions V1-V2 the target primers 27F—AGAGTTTGATCCTGGCTCAG and 375R—CTGCTGCCTYCCGTA were used with Illumina adapters for indexing PCR reaction according to Illumina's 16 s metagenomic sequencing library preparation guide (http://www.illumina.com/content/dam/illumina-support/documents/documentation/chemistry_documentation/16s/16s-metagenomic-library-prep-guide-15044223-b.pdf; last accessed July 2017). Cycling conditions were of initial denaturation at 95°C for 3 min followed by 30 cycles of denaturation at 95°C for 45 s, annealing of primers at 55°C for 45 s and extension at 72°C for 1 min followed by a final extension step at 72°C for 7 min and subsequent cooling to 4°C. Triplicates were pooled and checked on a 1% agarose gel before normalization of 20 μl PCR product on a SequalPrep Normalization Plate according to manufacturer's instructions (LifeTechnologies, Germany). Fifteen microliter of the normalized PCR product were used as template in a single 50 μl indexing PCR reaction for 8 cycles; the cycling conditions were as described above for the targeted PCR. Five microliter PCR product from each sample were pooled to the final sequencing library and 30 μl of the unpurified library were loaded to a 1% agarose gel for purification with the QIAquick gel extraction kit (Qiagen, Hilden, Germany) according to manufacturer's instructions. The purified library was quantified with QuantiFluor ONE dsDNA Dye on Quantus™ Fluorometer (Promega, Mannheim, Germany), loaded to an Agilent BioAnalyzer 2100 (Waldbronn, Germany) for quality control and the 6 pM library was sequenced on a MiSeq desktop sequencer (Illumina, Eindhoven, Netherlands) containing 20% PhiX control DNA (Illumina, Eindhoven, Netherlands) with v2 chemistry for 500 cycles according to manufacturer's instructions. FastQ raw reads were used for subsequent data analysis.

### Data analysis

A total of 2,825,234 MiSeq paired-end raw sequence forward and reverse reads were merged using ea-utils v1.1.2 (Aronesty, 2013) with standard settings, followed by a split library step from the Quantitative Insights Into Microbial Ecology (QIIME, v1.9.1) software (Caporaso et al., 2010). During this step sequence reads shorter than 200 nucleotides, reads that contain ambiguous bases or reads with an average quality score less than 30 were discarded. Chimera were removed with USEARCH v6.1 method in QIIME against 97% clustered GreenGenes reference 16S rRNA gene database (v13.8). In the second step OTU picking was done with QIIME open reference pipeline performing clustering steps at 97% sequence similarity, the taxonomy assignment with UCLUST algorithm (Edgar et al., 2011), alignment of reference sequences with pyNAST and generation of phylogenetic tree with FastTree. Finally, the OTU table was reduced by removing all OTUs present in only one sample with less than 10 reads. Downstream statistical analysis was performed in R version 3.3.3 (R Core Team, 2016) using a custom script. Principal Component Analysis (PCA) was performed using the prcomp function with default parameters. *P*-values for the group overlap in the PCA were calculated according to Goodpaster (Goodpaster and Kennedy, 2011) and Worley (Worley et al., 2013).

## Results

A total of 30 patients were included in the present study that underwent routine diagnostic gastroscopy, were never treated for H.p. infection before and did not have any gastroscopic investigation in the previous ten years (Table 1). The cohort included patients with gastric biopsies that tested negative for H.p. by histopathology and H.p.-specific PCR (*n* = 10, group 1), positive for H.p. by histopathology and H.p.-specific PCR but negative by CagA-specific PCR (*n* = 10, group 2), or positive in all three assays (*n* = 10, group 3). The mean age of the 30 patients was 50 years (*SD* = 16.4 years; range, 23–83 years) and 53% (16 of 30) of them were female. Gastroscopy revealed pathological findings in 24 (80%) patients, including gastritis in 20 patients (67%), gastro-esophageal reflux disease in 8 patients (27%), and abdominal hernia in 4 patients (13%). During data analysis, patient P004Z10, who belonged to the H.p.- group 1, was removed from further analysis because of a history of gastric paresis due to poorly controlled diabetes mellitus Typ I. An indication for this clinical diagnosis was the very high relative abundance of the family *Enterobacteriaceae* with 96.43% in the sample that might has been possible to migrate from the gut to the gastric lumen due to the disruption of the stomach's barrier function. The patient P004Z10 was removed from any further data analysis ending up with 9 samples for the H.p.- group. NGS data revealed highest accordance to the qRT-PCR and histopathology data in sample classification with 0–2.91% of H.p. reads in the H.p.- sample group 1 compared to 13.67–99.31% H.p. reads in the H.p.+/CagA− sample group 2 and 78.41–98.47% in the H.p.+/CagA+ sample group 3.

**Table 1:** Samples' H.p. and CagA status, patient age and sex, indication and the diagnosis for gastroscopy and histopathological score (^**^score).

**Sample ID**	Reads^*^	**H.p./CagA**	**Age**	**Sex**	**indication**	**diagnosis**	Score^**^
P005-Z08	11346	H.p.−	23	F	cd	nf	na
P002-Z18	11460	H.p.−	38	F	rs	g, r	na
P003-Z10	13651	H.p.−	56	M	uap	g	na
P001-Z14	17305	H.p.−	56	F	uap	ci	na
P004-Z10^e^	18310	H.p.−	83	M	bl	g, r, u	na
P001-Z12	29111	H.p.−	54	M	uap	g, r, öv	na
P007-Z08	35447	H.p.−	50	M	uap	r	na
P001-Z13	36680	H.p.−	66	M	rs	nf	na
P004-Z17	39276	H.p.−	27	M	uap	nf	na
P001-Z18	45063	H.p.−	50	M	un	r	na
P052-Z10	15743	H.p.+/CagA−	50	F	un	g	3+
P107-Z15	16451	H.p.+/CagA−	51	M	rs	g, r, he	2+
P033-Z12	26728	H.p.+/CagA−	66	F	un	g, r	1+
P008-Z17	26941	H.p.+/CagA−	34	F	uap	nf	2+
P058-Z14	27627	H.p.+/CagA−	43	M	rs	g, ci	3+
P080-Z15	44882	H.p.+/CagA−	76	F	uap	g, he	2+
P003-Z15	48255	H.p.+/CagA−	69	F	cu	g	2+
P022-Z12	48502	H.p.+/CagA−	75	F	un	g, he, di	3+
P066-Z10	57636	H.p.+/CagA−	64	M	bl	g, he	2+
P022-Z13	188261	H.p.+/CagA−	71	F	cu	g	2+
P121-Z15	11223	H.p.+/CagA+	33	M	uap	g	2+
P029-Z13	13309	H.p.+/CagA+	37	F	rs	nf	3+
P047-Z15	13310	H.p.+/CagA+	40	M	uap	g, ud	2+
P060-Z15	16297	H.p.+/CagA+	59	F	rs	g, r	2+
P062-Z14	20451	H.p.+/CagA+	51	F	rs	ci	3+
P021-Z12	27768	H.p.+/CagA+	21	F	ibs	g	3+
P008-Z08	33557	H.p.+/CagA+	32	M	uap	g	3+
P022-Z17	59142	H.p.+/CagA+	50	M	an	nf	2+
P061-Z10	73327	H.p.+/CagA+	32	F	ibs	g, di, l	3+
P045-Z10-A	79271	H.p.+/CagA+	51	M	bl	g	3+

* *Total Reads after quality filtering but before rarefaction to 11.200 reads for Qiime analysis. na: not applicable. e: not included to statistical analysis. Indication: an: anemia, bl: bleeding, cd: celiac disease, cu: check-up, ibs: irritable bowel syndrome, rs: reflux symptoms, uap: upper abdominal pain, un: unknown. Diagnosis: ci: cardiac insufficiency, di: duodenal inflammation, g: gastritis, he: hernia, l: lymphoedema, nf: no finding, öv: ösophagus varizen, r: reflux, u: ulcer, ud: ulcer duodenum*.

### Microbiological analysis

**Figure 1:**
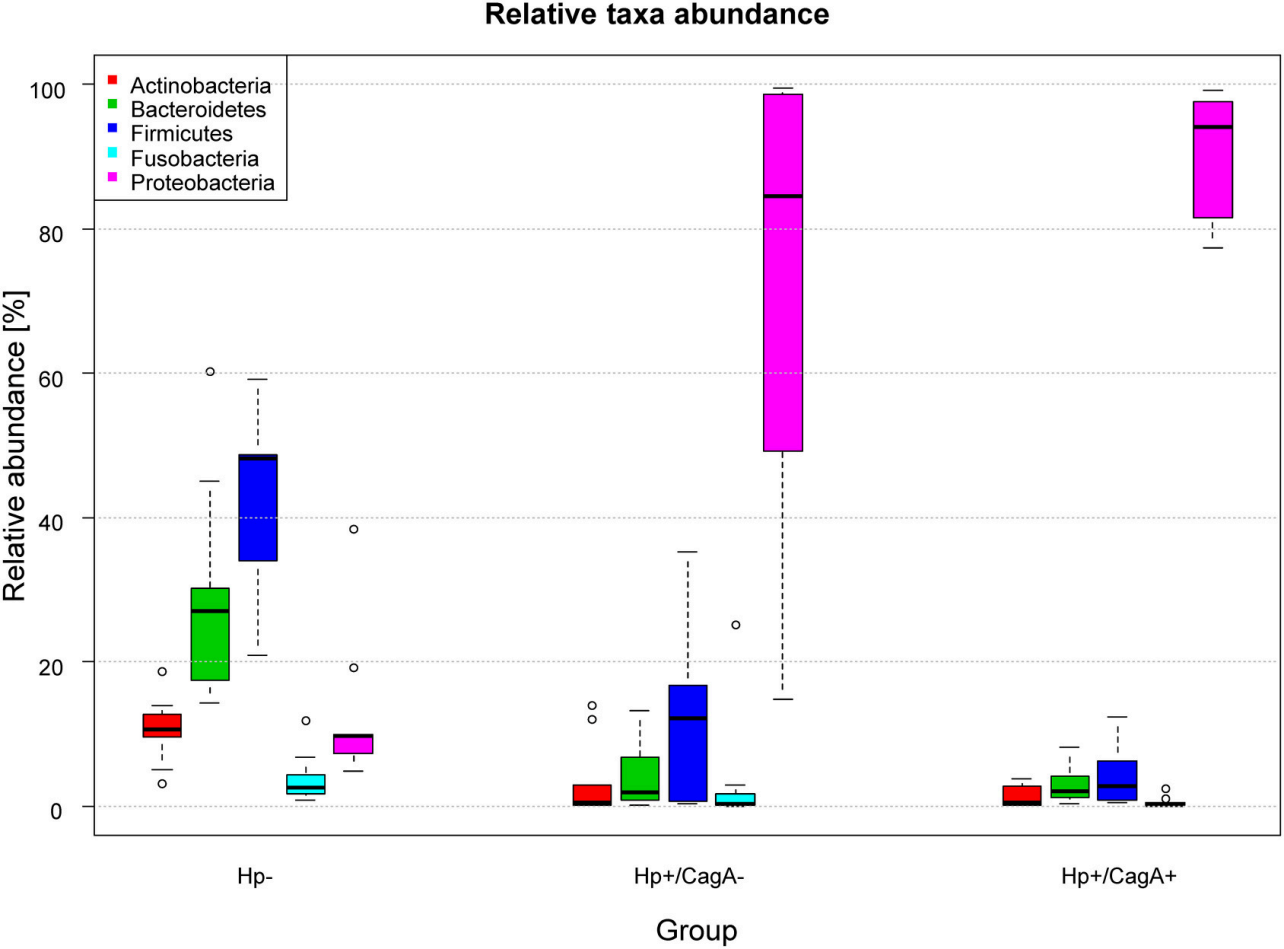
Box plot of the median relative abundances of the most dominant phyla in the three sample groups (*Firmicutes, Bacteroidetes, Proteobacteria, Actinobacteria*, and *Fusobacteria*). The phylum *Proteobacteria* (with the genus *Helicobacter*) increased in its relative abundance from the H.p.- over the H.p.+/CagA− to the H.p.+/CagA+ group. Significant differences were only found between the H.p.− vs the H.p.+/CagA− and the H.p.− vs H.p.+/CagA+ groups, respectively.

16S rRNA gene amplicon analysis of total DNA isolated from gastric biopsies yielded in average 37.518 reads per sample over all groups (range 11.223–188.261). All FastQ raw data may be accessed through the SRA accession number PRJEB22107 at the European Nucleotide Archive (ENA). The 29 samples used for data analysis (as patient P004Z10 was removed from the initial dataset of 30 samples) were grouped according their H.p. and CagA status into groups of 9 H.p.−, 10 H.p.+/CagA−, and 10 H.p.+/CagA+ samples (Table 1). The median number of reads per sample differed between the three groups and ranged between 29.111 (IQR = [13.651, 36.680]) in group H.p.−, 36.255 (IQR = [26.728, 48.502]) in the H.p.+/CagA− group and 24.110 (IQR = [13.310, 59.142]) in the H.p.+/CagA+ group. Sample P022-Z13 was an outlier with 188.261 reads in the H.p.+/CagA− group) (Supplementary Figure 1). After rarefaction of all samples to 11.200 reads per sample, a total number of 1208 operational taxonomical units (OTUs) was found ranging from 768 OTUs in the H.p.- group, 769 OTUs in the H.p.+/CagA− group, and 543 OTUs in the H.p.+/CagA+ group. Relative abundance of OTUs revealed that overall gastric biopsy samples were dominated by the phyla *Proteobacteria* (60.0%), *Firmicutes* (19.80%), *Bacteroidetes* (11.86%), *Actinobacteria* (4.47%), and *Fusobacteria* (2.53%). In the H.p.- group, the microbiome was diverse and dominated by *Firmicutes* (42.58%), *Bacteroidetes* (29.19%), *Proteobacteria* (12.82%), *Actinobacteria* (10.59%), and *Fusobacteria* (3.79%) (Figure 1) corresponding to former published results (Brawner et al., 2014; Lertpiriyapong et al., 2014; Patel et al., 2016; Schulz et al., 2016b). Upon H.p. infection, *Proteobacteria* increased dramatically to the most abundant microbial phylum (median relative abundance: H.p.+/CagA− = 84.55%, H.p.+/CagA+ = 94.05%; Figure 1). Nevertheless, statistically significant differences in the relative abundances of microbes on phylum level were detected only between the H.p.-negative and Hp+/CagA− and H.p.+/CagA+ group, respectively but not between the Hp+/CagA− vs. Hp+/CagA+ groups (two-sided Mann-Whitney *U*-test, adj.p-values see Supplementary Table 1). *Actinobacteria, Bacteroidetes, Firmicutes*, and *Proteobacteria* revealed statistical significant differences between the H.p.− vs. H.p.+/CagA− (adj.p-value = 0.0094, adj.p-value = 0.0019, adj.p-value = 0.0003 and adj.p-value = 0.0003, respectively) and the H.p.− vs. H.p.+/CagA+ groups (adj.p-value = 0.0006, adj.p-value = 0.0017, adj.p-value = 0.0001 and adj.p-value < 0.000). The relative abundance of *Fusobacteria* was altered significantly between the H.p.− vs. H.p.+/CagA+ groups (adj.p-value = 0.0375) (Supplementary Table 1). Presence of the CagA gene was not associated with significant differences in the relative abundances of the main phyla in H.p. positive biopsy samples (H.p.+/CagA− vs. H.p.+/CagA+), neither with significant differences of taxa in any other hierarchical level analyzed from class to genus level (Supplementary Table 1).

Class level analysis revealed that *Proteobacteria* of the H.p.- group included *Betaproteobacteria* and *Gammaproteobacteria* in equal rations whereas *Epsilonproteobacteria* dominated the H.p.+/CagA− as well as the H.p.+/CagA+ sample groups by far. Further, *Bacilli, Clostridia* and *Erysipelotrichi* were common taxa in all sample groups. The common orders *Lactobacillales* (30.14%) and *Bacteroidales* (24.09%) as well as *Actinomycetales* from H.p- samples were overgrown upon H.p. infection. *Lactobacillales* were dominated by the family *Streptococcaceae* in all sample groups. Further, H.p.+/CagA− samples were dominated by *Helicobacter* (81.99%) and *Streptococcus* (3.36%) and H.p.+/CagA+ by *Helicobacter* (93.21%), *Streptococcus* (1.04%), and *Prevotella* (1.03%). From the nine H.p. negative samples analyzed five were dominated by *Streptococcus* sp., three by *Prevotella* sp. and one by *Enterococcus* (*cecorum*). For more detailed information on tha distribution of specific taxa see also Supplementary Table 2. If applicable we performed species classification of the sequencing reads. The genus *Streptococcus* contained the species *Str. alactolyticus, Str. anginosus, Str. infantis*, and *Str. sobrinus*. The genus *Veillonella* contained the species *V. dispar* and *V. parvula* and the genus *Prevotella* the species *P. copri, P. intermedia, P. melaninogenica, P. nanceiensis*, and *P. nigrescens*. All analyzed *Helicobacter* reads belonged to the species *H. pylori* (Supplementary Table 2).

**Figure 2:**
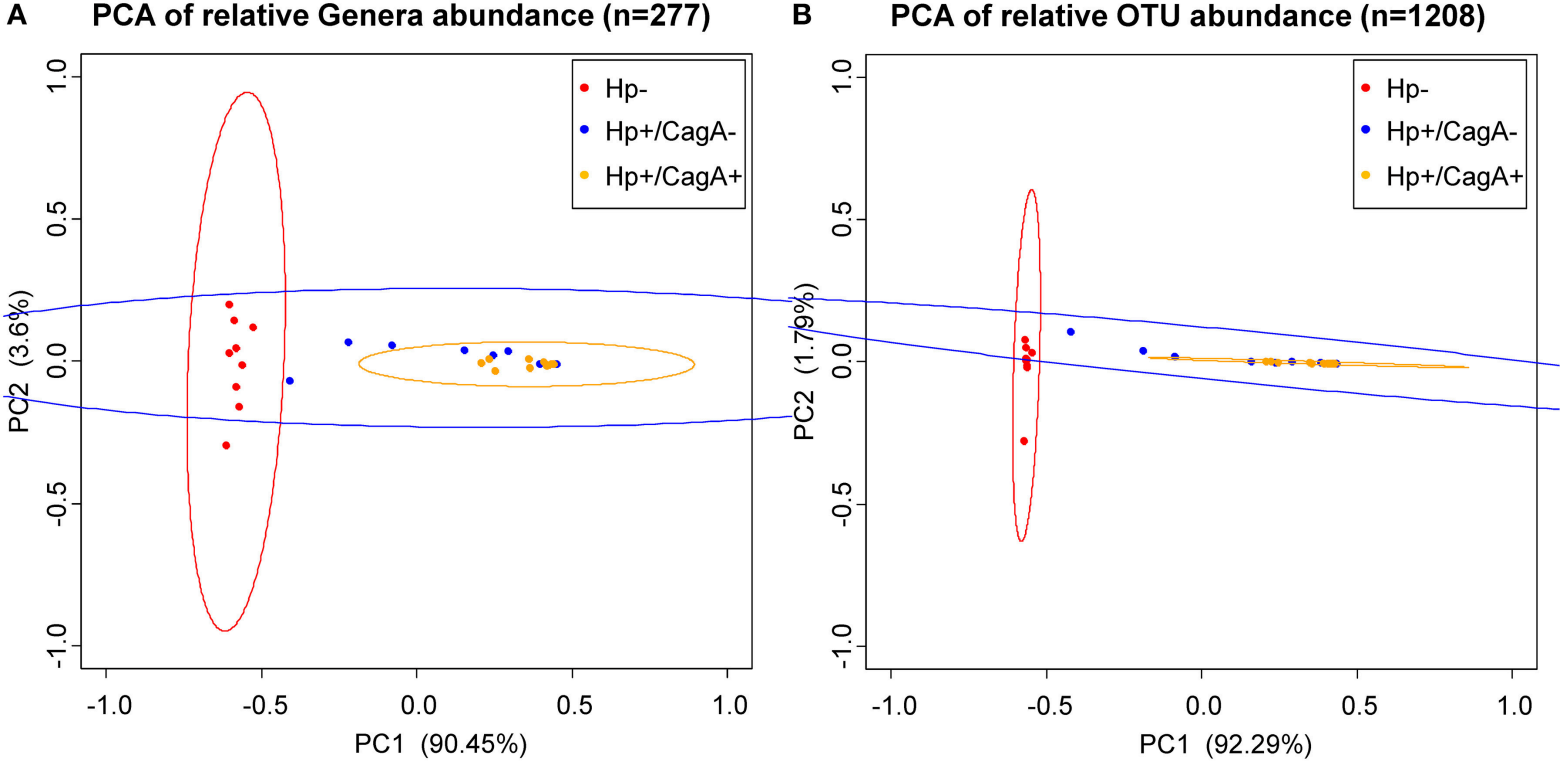
Principal Component Analysis (PCA) ordination plots of relative abundance of genera **(A)** and OTUs **(B)**. Statistically significant differences were found between H.p.− and the H.p.+/CagA− (adj. p-value OTU = 0.000001, adj. p-value genera = 0.000001) as well as between the H.p.− and the H.p.+/CagA+ groups (adj. p-value OTU = 0.000009, adj. p-value genera = 0.000023). No statistically significant differences were found between H.p.+/CagA− vs. H.p.+/CagA+ sample groups (adj. p-value OTU = 0.236619, adj. p-value genera = 0.095089). Ellipses denote the 95% confidence.

Principal Component Analysis (PCA) on OTU and genus level data revealed a clustering (means higher similarity of samples within the group than to the samples of other groups) of gastric samples according to H.p. status with a significant difference between the H.p. negative and the H.p.+/CagA+ group (adj. p-value OTU = 0.000001, adj. p-value genera = 0.000001) and the H.p. negative and the H.p.+/CagA− group (adj. p-value OTU = 0.000009, adj. p-value genera = 0.000023), but no statistically significant differences were found between the H.p.+/CagA− vs. H.p.+/CagA+ group (adj. p-value OTU = 0.236619, adj. p-value genera = 0.095089) (Figure 2).

### Effect of H.p. infection and CagA status on alpha diversity results of gastric samples

**Figure 3:**
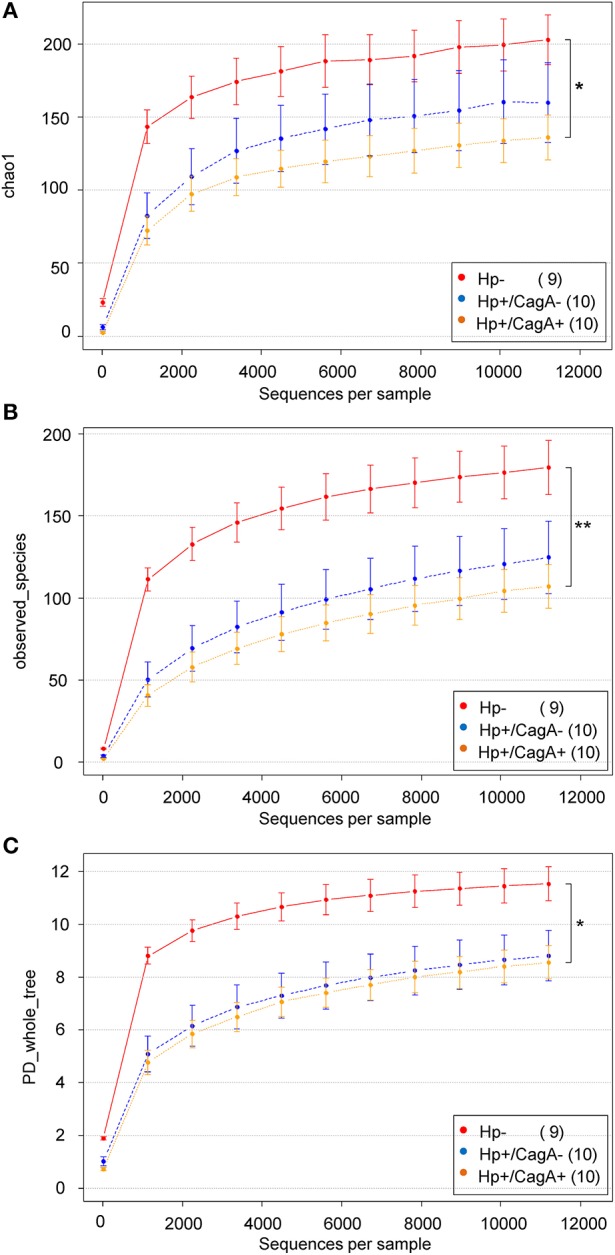
**(A)** Chao1, **(B)** PD-wholetree, and **(C)** observed species rarefied alpha diversity of the three sample groups (H.p.−, H.p.+/CagA−, H.p.+/CagA+). A significant difference was found between the H.p. negative and the H.p.+/CagA+ groups with a not significant intermediate phenotype of the H.p.+/CagA− group. Error bars denote standard error.

Three different alpha diversity measures were used on the rarefied sequences to analyze the microbial diversity and compare the results of different methods of calculation. The highest estimated richness (Chao1) of stomach microbiota was found in the H.p.- sample group with 203 compared to 160 in the H.p.+/CagA− and 136 in the H.p.+/CagA+ group (Figure 3A). Correspondingly, observed species as well as PD whole tree revealed the highest microbial diversity in H.p.− samples followed by the H.p.+/CagA− and the H.p.+/CagA+ group (Figures 3B,C). A decreasing alpha diversity from H.p.− to H.p.+/CagA− to H.p.+/CagA+ was observed but did not gain statistically significance (Figures 3A–C). All performed alpha diversity measures (Chao1, PD whole tree, and observed species), that were used to compare different methods of calculation, revealed significant differences between the H.p.− vs. H.p.+/CagA+ group (Table 2). Chao1 analysis revealed statistically significant differences between the H.p.− and the H.p.+/CagA+ groups (adj.p-value = 0.028) but no significant differences in estimated richness between the other comparators (H.p.− vs. H.p.+/CagA− adj.p-value = 0.402; H.p.+/CagA− vs. H.p.+/CagA+ adj.p-value = 0.535) (Table 2).

**Table 2:** Alpha diversity calculations for Chao1, observed species and PD whole tree.

**Type**	**Comparison**	***p*-value**	**adj.*p*.value**
chao1	H.p.– vs. H.p.+/CagA−	0.201	0.402
chao1	H.p.– vs. H.p.+/CagA+	0.009	0.028^*^
chao1	H.p.+/CagA- vs. H.p.+/CagA+	0.535	0.535
observed_species	H.p.– vs. H.p.+/CagA−	0.067	0.133
observed_species	H.p.– vs. H.p.+/CagA+	0.003	0.009^**^
observed_species	H.p.+/CagA- vs. H.p.+/CagA+	0.507	0.507
PD_whole_tree	H.p.– vs. H.p.+/CagA−	0.026	0.052
PD_whole_tree	H.p.– vs. H.p.+/CagA+	0.004	0.013^*^
PD_whole_tree	H.p.+/CagA- vs. H.p.+/CagA+	0.943	0.943

Significant differences were found in the H.p.– vs. H.p.+/CagA+ comparison.

* p-value < 0.05,

** *p-value < 0.01*.

Power calculations were performed to estimate the samples size required for statistically significant differences in the alpha diversity of all sample groups (Supplementary Table 3). Dependent on the alpha diversity measure, 257 to 21.262 specimens per group were estimated for significant differences between the H.p.+/CagA− vs. H.p.+/CagA+ sample groups (Supplementary Table 2). For significant differences between H.p.- vs. H.p.+/CagA− groups 19 to 67 samples per group were estimated (Supplementary Table 3).

### Distribution pattern of overlapping otus and genera in the three sample groups

**Figure 4:**
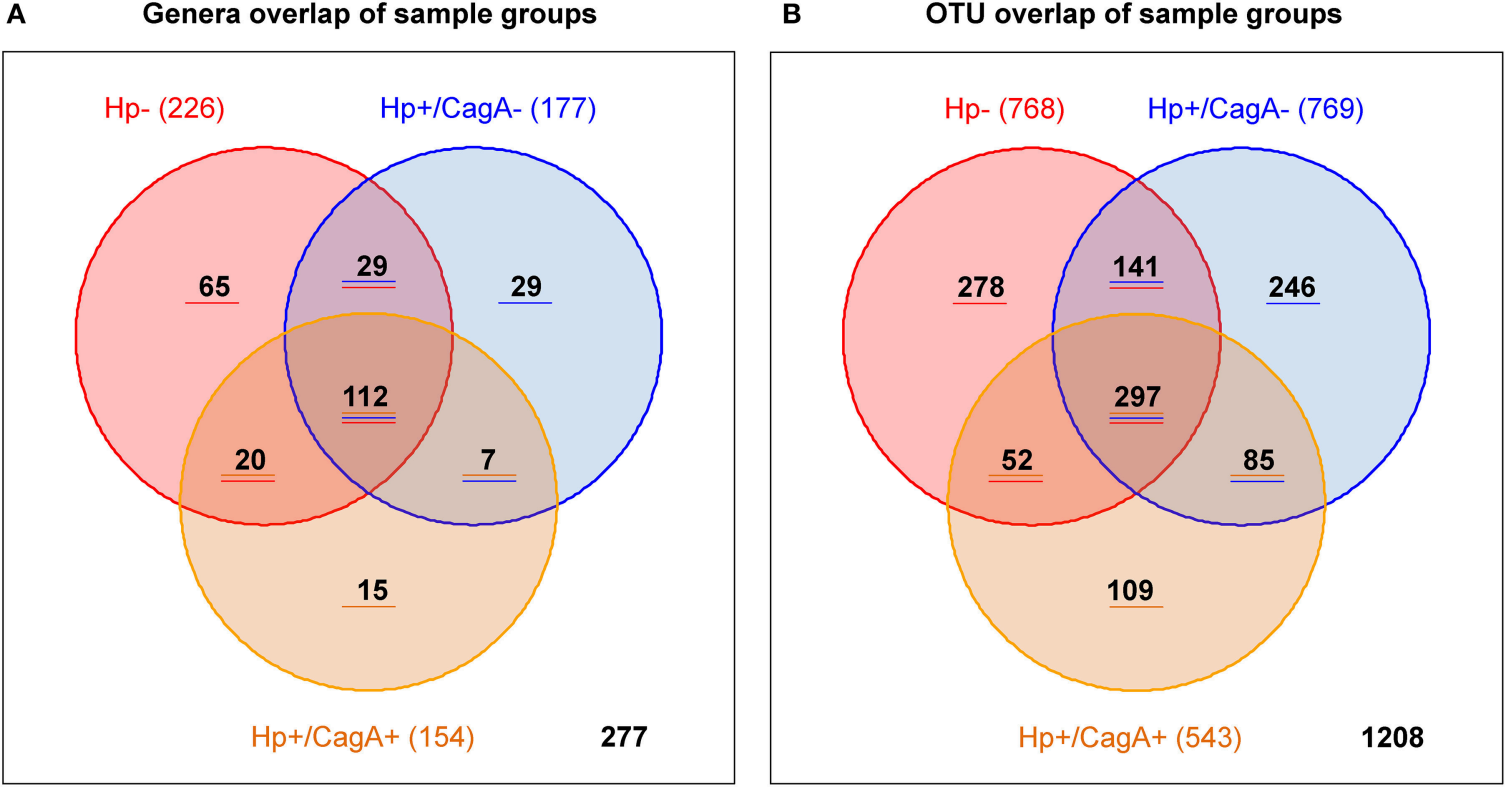
Venn diagram visualizing the number of group specific genera **(A)** and OTUs **(B)**.

Overlap analysis on genus and OTU level was performed with Venn diagrams to provide the number of genera and OTUs specific for one, two or all investigated gastric sample groups (Figures 4A,B). From the 226 genera found in H.p.- samples 112 were in common in all three sample groups, 65 were specific for the H.p.- group, 29 in common with the with the H.p.+/CagA− group and 20 in common with the H.p.+/CagA+ sample group (Figure 4A). 7 genera were in common between the H.p.+/CagA− and the H.p.+/CagA+ group and 29 specific for H.p.+/CagA− and 15 specific for H.p.+/CagA+ (Figure 4A and Supplementary Table 4). Filtering the genera for those that occur in at least 50% of the samples within one group with a relative abundance of at least 1% only 13 genera common for all sample groups were left, demonstrating that those genera responsible for the biological information are common in all sample groups. The group specific genera reveal less than 2% of the reads with exception of the sample P002Z18 in which genera specific for the H.p.- group reveal 10% of all reads in that sample. Similar distributions were found for the OTU pattern between the three groups (Figure 4B).

### Taxa significantly altered between the three sample groups

**Figure 5:**
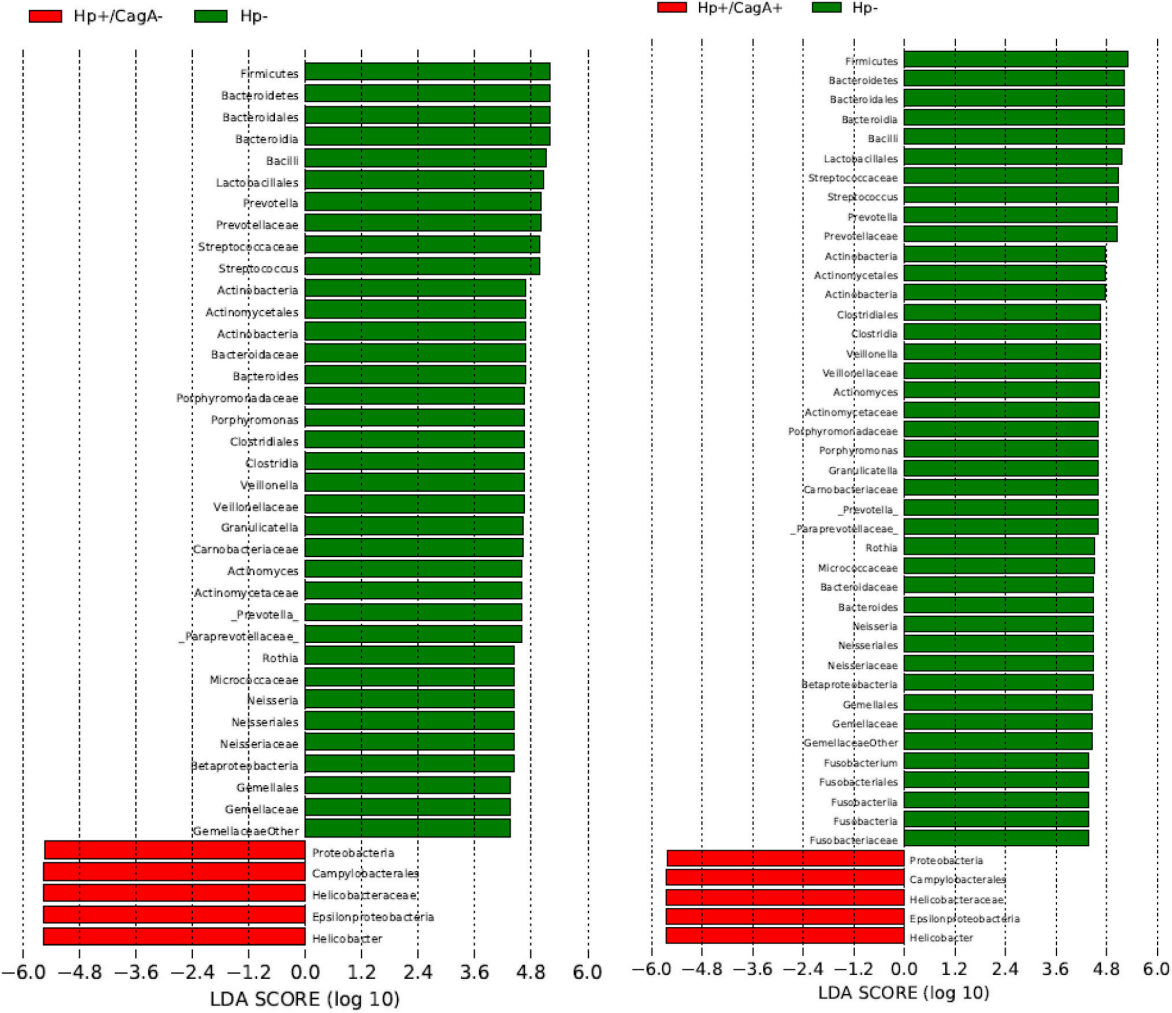
Linear discriminant Effect Size analysis (LefSe) analysis revealed over 40 taxa (H.p.− vs. H.p.+/CagA+ 45, H.p.− vs. H.p.+/CagA− 41) significantly different in their relative abundances between the sample groups in the pairwise comparisons H.p.− vs. H.p.+/CagA+, H.p.− vs. H.p.+/CagA− and H.p.+/CagA− vs. H.p.+/CagA+. Only taxa with a relative abundance of at least 1% in at least 50% of all samples out of one sample group were considered.

Linear discriminant Effect Size analysis (LefSe) was performed to identify those taxa significantly different in their relative abundances in pairwise comparisons of the three sample groups from phylum to species level (Segata et al., 2011). Only taxa with a relative abundance of at least 1% in at least 50% of the samples within one group were considered. The comparison of H.p.− vs. H.p.+/CagA+ revealed significant differences in 46 taxa, 41 taxa in H.p.− vs. H.p.+/CagA− and no taxa at all were found comparing samples from the H.p.+/CagA− vs. H.p.+/CagA+ groups (Figure 5). Significant alterations were detected at all hierarchical levels the genus *Fusobacterium* belongs to in the H.p.− vs. H.p.+/CagA+ but not in the H.p.− vs. H.p.+/CagA− comparison (Figure 5).

## Discussion

In this exploratory study on the characteristics of the human gastric microbial pattern and its relation to H.p. infection with and without detection of the CagA gene, we analyzed gastric biopsies from a cohort of 30 patients who were prospectively evaluated for H.p. in a prospective, clinical, multicenter trial. Former studies on the human gastric microbiome indicate a distinct gastric microbial pattern with *Actinobacteria, Bacteroidetes, Firmicutes*, and *Proteobacteria* as the dominating phyla and *Streptococcus* as the most dominant genus (Andersson et al., 2008; Khosravi et al., 2014; Ianiro et al., 2015; Llorca et al., 2016; Schulz et al., 2016a,b; Yang et al., 2016). Taxa identified in our data set correspond to these former studies from phylum to genus level. In concordance with our findings, culture dependent approaches found predominantly *Streptococcus, Neisseria, Klebsiella*, and *Lactobacillus* (Khosravi et al., 2014) with *Streptococcus* (8.61% over all samples) and *Neisseria* (1.13% over all samples) confirmed as dominant taxa. This study is distinguishing due to its prospective design, multicenter sample collection and the availability of numerous clinical data of the probands.

In the group of H.p. negative samples, as classified by negative test results in histopathology and qRT-PCR, only two of nine samples were totally free of H.p. reads and one with 0.003% relative abundance, less than one read at a rarefaction level of 11,200 reads per sample. H.p.-reads were found in the other six samples in a range of 0.02–2.9% relative abundance of reads. Contamination of samples may be ruled out because all negative control samples were totally free of H.p. reads at the mentioned rarefaction level. Nevertheless, upon detection of the H.p. infection by histopathology and qRT-PCR the relative abundances of other taxa detected by 16S rRNA gene based next generation sequencing methods decreased dramatically. Probably other taxa were still present but a much higher sequencing depth would be required to detect other sequences due to the disproportionately high amounts of H.p. DNA.

H.p.+/CagA+ samples were not associated with a significantly different gastric microbiome compared to H.p.+/CagA− ones in concordance with previous studies (Yang et al., 2016). Nevertheless, in the H.p.+/CagA− sample group, the relative abundance of H.p. was below 50% in three of the 10 samples (13, 37, and 47%, respectively) in contrast to the 10 samples in the H.p.+/CagA+ group that all showed an H.p. relative abundance of at least 70% or higher. Correspondingly, alpha diversity indices of the H.p.+/CagA− samples were increased compared to H.p.+/CagA+ ones, although not statistically significant with the analyzed specimen number. Increasing the sample number might result in significant differences between the Hp+/CagA+ and the H.p.+/CagA− samples group. Therefore, we hypothesize that CagA expression might be one of the virulence factors responsible for the successful proliferation of H.p. in the gastric habitat and the overgrowth of H.p. compared to other microorganisms.

### *Streptococcus sp*. in gastric samples

*Streptococcus* was frequently detected in the gastric mucosa of healthy, H.p. negative individuals as well as in H.p. positive ones (Khosravi et al., 2014; Ianiro et al., 2015). The close association between *Streptococci* and H.p. was further underlined by the observation that *Streptococcus mitis* interacted with H.p. upon co-cultivation by altered protein biosynthesis in H.p. (Khosravi et al., 2016) although not validated under native, acidic conditions. Additionally, *Streptococcus* exhibits urease activity, indicating an adaptive capability of *Streptococci* to the acidic conditions of the gastric environment (Chen et al., 2000). In our H.p. negative sample cohort, *Streptococcus* had a median relative abundance of 14.25% (*SD* = 10.91), with decreasing abundance in H.p.+/CagA− samples to 3.37% (*SD* = 7.50) and to 1.03% in the H.p.+/CagA+ sample group (*SD* = 2.02). Interestingly, three of the four *Streptococcus* species found in our gastric samples (*Str. anginosus, Str. infantis, Str. sobrinus*) have been reported also in the Human Oral Microbiome Database (HOMD) (Chen et al., 2010). The relative abundance of the genus *Streptococcus* was significantly reduced in both H.p. positive (Hp+/CagA+ and H.p.+/CagA−) sample groups compared to the H.p. negative group (adj.p-value 0.0216 and 0.0100 respectively). No significant difference in the relative abundance of *Streptococcus* was found between the H.p.+/CagA− vs. H.p.+/CagA+ group (adj.p-value, 0.1716). Hence, the CagA gene expression did not seem to impact *Streptococcus* gastric colonization. The relevance of co-localization of high abundant H.p. and *Streptococcus* for cancerogenesis (e.g., sample P008-Z17: H.p. 71.95% and *Str*. 13.61%; sample P080-Z15: H.p. 37.03% and *Str*. 24.04%) needs to be evaluated in detail.

### Relative abundance of H.p. in human gastric samples

Our data on the comparison of H.p. infected and not infected human gastric mucosa samples indicated a “takeover” of H.p in the gastric niche. H.p. negative samples showed a latent infection over 1% of relative abundance in two out of nine samples that did for some reasons not spread till sampling. Only two samples were totally free of H.p. reads and in five samples the relative abundance was far less than 1%. The relative abundance of non-H.p. taxa from H.p. negative samples was much higher compared to H.p. infected samples without and with CagA gene. Bacterial reads other than H.p. seemed to disappear in the H.p. samples possibly caused by insufficient coverage or biological competition.

### Overlapping genera in the three sample groups

The 65 genera found to be unique in the H.p.- group represented less than 2% of the total reads in these samples. Only in one of the H.p.- samples the unique genera represented 10.48% of all reads. The genera *Oxalobacteraceae* and *Methylophilaceae* were the most dominant taxa unique for the H.p.- groups, due to the low biological mass a protective function against H.p. infections was doubted. The same was observed for the 29 genera unique for the H.p.+/CagA− group as well as for the 15 genera unique for the H.p.+CagA+ group representing less than 1% of all samples reads questioning their biological relevance. Thus, biological changes detected were caused basically by the 13 most dominant taxa common for all three sample groups.

### Technical limitations of gastric microbiome studies

The human gastric microbiome is more and more under the focus of clinical research (Majlessi et al., 2017; Noto and Peek, 2017; Péré-Védrenne et al., 2017; Shah, 2017). Recent studies on the oral, gastric and duodenal microbiome describe no significant differences at phylum level between the oral habitat and the stomach aspirates but clear differences compared to the duodenal phyla (Schulz et al., 2016a). In our opinion this is hard to believe as the oral habitat differs from the gastric so dramatically in environmental conditions. Instead, technical limitations must be considered in culture-dependent as well as culture independent approaches. DNA based studies detected all present nucleic acids. Under the permanent process of swallowing microbiota inhabiting the oral cavity or food derived microbial DNA are characterized as well. In studies on cultivable bacterial microbiota of the human stomach, samples are spread to conventional media (Vega et al., 2003) without adapting the culture conditions to those present in the natural gastric habitat (Sanduleanu et al., 2001; Delgado et al., 2013; Khosravi et al., 2014). We suggest that gastric habitat like conditions would be inevitable for serious conclusions on viability and above all proliferation capability of isolated microorganisms. In the same manner, characterization of human gastric microbiota in patients with acid inhibitors does not reflect natural stomach conditions and cannot exclude oral or intestinal sources overgrowing the natural gastric community due to the artificially increased stomach pH and therefore the disruption of its barrier function (Paroni Sterbini et al., 2016; Llorente et al., 2017) putting their physiological relevance in the healthy, acidic gastric lumen into question. Finally, the source of contamination of gastric biopsy samples through the gastroscopic instrument and sampling procedure from the upper oropharyngeal region needs to be considered.

A method to discriminate between live and dead cells would be the treatment of freshly collected samples with Propidium Monoazide (PMA) and thereby masking cell free DNA derived from dead microorganisms for PCR reaction (Nocker et al., 2007). Nevertheless, we hypothesize that this approach as well as the approach to work with isolated RNA instead of DNA (Schulz et al., 2016a) does not represent an improvement as saliva is swallow continuously, therefore living microorganisms are delivered to the stomach continuously and swallowed microorganisms may tolerate the acidic conditions without proliferation for a certain period of time.

In conclusion, we describe a distinct microbial pattern of the human gastric microbiome from biopsy samples of a clinically well characterized cohort collected at eight different geographic locations in Austria. We observe a dramatic decrease of the 16S rRNA gene based detected non H.p. bacterial microbiome in H.p. infected samples and from our results, presence of the CagA gene has no statistically significant influence on accompanying microbial taxa, although a non-significant trend on the microbial alpha diversity is observed.

## Author contributions

IK: Study design, performed analysis, analyzed the data, wrote the manuscript. CB: Performed analysis, wrote the manuscript. AS: Performed analysis. JT: Performed analysis, wrote the manuscript. M-TK: Performed analysis. CH: Wrote the manuscript. AP: Performed analysis. SB-F: Performed analysis. CS-K: Performed analysis. GGT: Analyzed the data, wrote the manuscript. CS: Study design, performed analysis, analyzed the data, wrote the manuscript.

### Conflict of interest statement

The authors declare that the research was conducted in the absence of any commercial or financial relationships that could be construed as a potential conflict of interest.
