# Supplementary material for: The Human Gastric Microbiome Is Predicated upon Infection with Helicobacter pylori
Source: Front Microbiol. 2017 Dec 14;8:2508. doi: 10.3389/fmicb.2017.02508 (PMC5735373; doi:10.3389/fmicb.2017.02508)
Supplement: Supplementary file 1 [file Table1.DOCX]

**Supplementary Table 1:** Two-sided t-test and adj.pvalues of those taxa with significant differences in their relative abundances between the three sample groups from phylum to genus level. Significance: * adj.pvalue <0.05, ** adj.pvalue <0.01 and *** adj.pvalue <0.001.

**level taxon comparison mean1 mean2 pvalue adj.pvalue sig**

Phylum Actinobacteria Hp- vs. Hp+/CagA- 0.1060 0.0332 0.0050 0.0094 **

Phylum Actinobacteria Hp- vs. Hp+/CagA+ 0.1060 0.0113 0.0002 0.0006 ***

Phylum Actinobacteria Hp+/CagA- vs. Hp+/CagA+ 0.0332 0.0113 0.2293 0.2866

Phylum Bacteroidetes Hp- vs. Hp+/CagA- 0.2919 0.0430 0.0009 0.0019 **

Phylum Bacteroidetes Hp- vs. Hp+/CagA+ 0.2919 0.0294 0.0007 0.0017 **

Phylum Bacteroidetes Hp+/CagA- vs. Hp+/CagA+ 0.0430 0.0294 0.4564 0.4890

Phylum Firmicutes Hp- vs. Hp+/CagA- 0.4258 0.1247 0.0001 0.0003 ***

Phylum Firmicutes Hp- vs. Hp+/CagA+ 0.4258 0.0425 0.0000 0.0001 ***

Phylum Firmicutes Hp+/CagA- vs. Hp+/CagA+ 0.1247 0.0425 0.0714 0.1071

Phylum Fusobacteria Hp- vs. Hp+/CagA- 0.0380 0.0309 0.8002 0.8002

Phylum Fusobacteria Hp- vs. Hp+/CagA+ 0.0380 0.0045 0.0225 0.0375 *

Phylum Fusobacteria Hp+/CagA- vs. Hp+/CagA+ 0.0309 0.0045 0.3130 0.3612

Phylum Proteobacteria Hp- vs. Hp+/CagA- 0.1282 0.7375 0.0001 0.0003 ***

Phylum Proteobacteria Hp- vs. Hp+/CagA+ 0.1282 0.9109 0.0000 0.0000 ***

Phylum Proteobacteria Hp+/CagA- vs. Hp+/CagA+ 0.7375 0.9109 0.1030 0.1404

Class Actinobacteria Hp- vs. Hp+/CagA- 0.0994 0.0293 0.0055 0.0120 *

Class Actinobacteria Hp- vs. Hp+/CagA+ 0.0994 0.0101 0.0003 0.0014 **

Class Actinobacteria Hp+/CagA- vs. Hp+/CagA+ 0.0293 0.0101 0.2692 0.3288

Class Bacilli Hp- vs. Hp+/CagA- 0.3296 0.0913 0.0004 0.0014 **

Class Bacilli Hp- vs. Hp+/CagA+ 0.3296 0.0274 0.0001 0.0006 ***

Class Bacilli Hp+/CagA- vs. Hp+/CagA+ 0.0913 0.0274 0.0636 0.0955

Class Bacteroidia Hp- vs. Hp+/CagA- 0.2703 0.0402 0.0020 0.0061 **

Class Bacteroidia Hp- vs. Hp+/CagA+ 0.2703 0.0274 0.0016 0.0054 **

Class Bacteroidia Hp+/CagA- vs. Hp+/CagA+ 0.0402 0.0274 0.4759 0.5192

Class Clostridia Hp- vs. Hp+/CagA- 0.0917 0.0302 0.0035 0.0093 **

Class Clostridia Hp- vs. Hp+/CagA+ 0.0917 0.0145 0.0002 0.0012 **

Class Clostridia Hp+/CagA- vs. Hp+/CagA+ 0.0302 0.0145 0.2740 0.3288

Class Fusobacteriia Hp- vs. Hp+/CagA- 0.0380 0.0309 0.8002 0.8350

Class Fusobacteriia Hp- vs. Hp+/CagA+ 0.0380 0.0045 0.0225 0.0416 *

Class Fusobacteriia Hp+/CagA- vs. Hp+/CagA+ 0.0309 0.0045 0.3130 0.3578

Class Betaproteobacteria Hp- vs. Hp+/CagA- 0.0497 0.0060 0.0070 0.0140 *

Class Betaproteobacteria Hp- vs. Hp+/CagA+ 0.0497 0.0020 0.0045 0.0107 *

Class Betaproteobacteria Hp+/CagA- vs. Hp+/CagA+ 0.0060 0.0020 0.1341 0.1788

Class Epsilonproteobact. Hp- vs. Hp+/CagA- 0.0092 0.7252 0.0000 0.0004 ***

Class Epsilonproteobact. Hp- vs. Hp+/CagA+ 0.0092 0.9026 0.0000 0.0000 ***

Class Epsilonproteobact. Hp+/CagA- vs. Hp+/CagA+ 0.7252 0.9026 0.0972 0.1373

Class Gammaproteobact. Hp- vs. Hp+/CagA- 0.0595 0.0059 0.0300 0.0480 *

Class Gammaproteobact. Hp- vs. Hp+/CagA+ 0.0595 0.0056 0.0293 0.0480 *

Class Gammaproteobact. Hp+/CagA- vs. Hp+/CagA+ 0.0059 0.0056 0.8600 0.8600

Order Actinomycetales Hp- vs. Hp+/CagA- 0.0994 0.0293 0.0055 0.0139 *

Order Actinomycetales Hp- vs. Hp+/CagA+ 0.0994 0.0101 0.0003 0.0016 **

Order Actinomycetales Hp+/CagA- vs. Hp+/CagA+ 0.0293 0.0101 0.2692 0.3082

Order Bacteroidales Hp- vs. Hp+/CagA- 0.2703 0.0402 0.0020 0.0069 **

Order Bacteroidales Hp- vs. Hp+/CagA+ 0.2703 0.0274 0.0016 0.0060 **

Order Bacteroidales Hp+/CagA- vs. Hp+/CagA+ 0.0402 0.0274 0.4759 0.4942

Order Campylobacterales Hp- vs. Hp+/CagA- 0.0092 0.7252 0.0000 0.0004 ***

Order Campylobacterales Hp- vs. Hp+/CagA+ 0.0092 0.9026 0.0000 0.0000 ***

Order Campylobacterales Hp+/CagA- vs. Hp+/CagA+ 0.7252 0.9026 0.0972 0.1456

Order Clostridiales Hp- vs. Hp+/CagA- 0.0917 0.0302 0.0035 0.0105 *

Order Clostridiales Hp- vs. Hp+/CagA+ 0.0917 0.0145 0.0002 0.0014 **

Order Clostridiales Hp+/CagA- vs. Hp+/CagA+ 0.0302 0.0145 0.2740 0.3082

Order Fusobacteriales Hp- vs. Hp+/CagA- 0.0380 0.0309 0.8002 0.8002

Order Fusobacteriales Hp- vs. Hp+/CagA+ 0.0380 0.0045 0.0225 0.0434 *

Order Fusobacteriales Hp+/CagA- vs. Hp+/CagA+ 0.0309 0.0045 0.3130 0.3381

Order Gemellales Hp- vs. Hp+/CagA- 0.0353 0.0144 0.0874 0.1388

Order Gemellales Hp- vs. Hp+/CagA+ 0.0353 0.0026 0.0057 0.0139 *

Order Gemellales Hp+/CagA- vs. Hp+/CagA+ 0.0144 0.0026 0.1502 0.1932

Order Lactobacillales Hp- vs. Hp+/CagA- 0.2924 0.0692 0.0006 0.0026 **

Order Lactobacillales Hp- vs. Hp+/CagA+ 0.2924 0.0246 0.0002 0.0014 **

Order Lactobacillales Hp+/CagA- vs. Hp+/CagA+ 0.0692 0.0246 0.1241 0.1675

Order Neisseriales Hp- vs. Hp+/CagA- 0.0312 0.0056 0.0200 0.0416 *

Order Neisseriales Hp- vs. Hp+/CagA+ 0.0312 0.0012 0.0091 0.0205 *

Order Neisseriales Hp+/CagA- vs. Hp+/CagA+ 0.0056 0.0012 0.1025 0.1456

Order Xanthomonadales Hp- vs. Hp+/CagA- 0.0245 0.0016 0.0718 0.1292

Order Xanthomonadales Hp- vs. Hp+/CagA+ 0.0245 0.0022 0.0784 0.1323

Order Xanthomonadales Hp+/CagA- vs. Hp+/CagA+ 0.0016 0.0022 0.1955 0.2400

Family Actinomycetaceae Hp- vs. Hp+/CagA- 0.0526 0.0107 0.0049 0.0207 *

Family Actinomycetaceae Hp- vs. Hp+/CagA+ 0.0526 0.0064 0.0023 0.0140 *

Family Actinomycetaceae Hp+/CagA- vs. Hp+/CagA+ 0.0107 0.0064 0.5371 0.5639

Family Bacteroidaceae Hp- vs. Hp+/CagA- 0.0236 0.0017 0.0184 0.0387 *

Family Bacteroidaceae Hp- vs. Hp+/CagA+ 0.0236 0.0024 0.0211 0.0403 *

Family Bacteroidaceae Hp+/CagA- vs. Hp+/CagA+ 0.0017 0.0024 0.4560 0.5040

Family Carnobacteriaceae Hp- vs. Hp+/CagA- 0.0385 0.0073 0.0021 0.0140 *

Family Carnobacteriaceae Hp- vs. Hp+/CagA+ 0.0385 0.0036 0.0011 0.0119 *

Family Carnobacteriaceae Hp+/CagA- vs. Hp+/CagA+ 0.0073 0.0036 0.2270 0.2724

Family Fusobacteriaceae Hp- vs. Hp+/CagA- 0.0281 0.0297 0.9525 0.9525

Family Fusobacteriaceae Hp- vs. Hp+/CagA+ 0.0281 0.0019 0.0256 0.0468 *

Family Fusobacteriaceae Hp+/CagA- vs. Hp+/CagA+ 0.0297 0.0019 0.2879 0.3358

Family Gemellaceae Hp- vs. Hp+/CagA- 0.0353 0.0144 0.0874 0.1411

Family Gemellaceae Hp- vs. Hp+/CagA+ 0.0353 0.0026 0.0057 0.0217 *

Family Gemellaceae Hp+/CagA- vs. Hp+/CagA+ 0.0144 0.0026 0.1502 0.2036

Family Helicobacteraceae Hp- vs. Hp+/CagA- 0.0052 0.7238 0.0000 0.0007 ***

Family Helicobacteraceae Hp- vs. Hp+/CagA+ 0.0052 0.9021 0.0000 0.0000 ***

Family Helicobacteraceae Hp+/CagA- vs. Hp+/CagA+ 0.7238 0.9021 0.0981 0.1525

Family Neisseriaceae Hp- vs. Hp+/CagA- 0.0312 0.0056 0.0200 0.0400 *

Family Neisseriaceae Hp- vs. Hp+/CagA+ 0.0312 0.0012 0.0091 0.0226 *

Family Neisseriaceae Hp+/CagA- vs. Hp+/CagA+ 0.0056 0.0012 0.1025 0.1537

Family Micrococcaceae Hp- vs. Hp+/CagA- 0.0360 0.0179 0.2194 0.2710

Family Micrococcaceae Hp- vs. Hp+/CagA+ 0.0360 0.0031 0.0079 0.0226 *

Family Micrococcaceae Hp+/CagA- vs. Hp+/CagA+ 0.0179 0.0031 0.2027 0.2580

Family [Paraprevotellaceae] Hp- vs. Hp+/CagA- 0.0349 0.0030 0.0078 0.0226 *

Family [Paraprevotellaceae] Hp- vs. Hp+/CagA+ 0.0349 0.0038 0.0089 0.0226 *

Family [Paraprevotellaceae] Hp+/CagA- vs. Hp+/CagA+ 0.0030 0.0038 0.6404 0.6560

Family Porphyromonadac. Hp- vs. Hp+/CagA- 0.0258 0.0038 0.0087 0.0226 *

Family Porphyromonadac. Hp- vs. Hp+/CagA+ 0.0258 0.0012 0.0049 0.0207 *

Family Porphyromonadac. Hp+/CagA- vs. Hp+/CagA+ 0.0038 0.0012 0.1166 0.1664

Family Prevotellaceae Hp- vs. Hp+/CagA- 0.1855 0.0317 0.0113 0.0251 *

Family Prevotellaceae Hp- vs. Hp+/CagA+ 0.1855 0.0200 0.0077 0.0226 *

Family Prevotellaceae Hp+/CagA- vs. Hp+/CagA+ 0.0317 0.0200 0.4843 0.5215

Family Streptococcaceae Hp- vs. Hp+/CagA- 0.1900 0.0604 0.0098 0.0229 *

Family Streptococcaceae Hp- vs. Hp+/CagA+ 0.1900 0.0186 0.0014 0.0119 *

Family Streptococcaceae Hp+/CagA- vs. Hp+/CagA+ 0.0604 0.0186 0.1189 0.1664

Family Veillonellaceae Hp- vs. Hp+/CagA- 0.0667 0.0209 0.0034 0.0177 *

Family Veillonellaceae Hp- vs. Hp+/CagA+ 0.0667 0.0120 0.0004 0.0053 **

Family Veillonellaceae Hp+/CagA- vs. Hp+/CagA+ 0.0209 0.0120 0.3891 0.4417

Family Xanthomonadac. Hp- vs. Hp+/CagA- 0.0245 0.0016 0.0721 0.1261

Family Xanthomonadac. Hp- vs. Hp+/CagA+ 0.0245 0.0022 0.0787 0.1323

Family Xanthomonadac. Hp+/CagA- vs. Hp+/CagA+ 0.0016 0.0022 0.1955 0.2566

Genus Actinomyces Hp- vs. Hp+/CagA- 0.0526 0.0107 0.0049 0.0190 *

Genus Actinomyces Hp- vs. Hp+/CagA+ 0.0526 0.0064 0.0023 0.0129 *

Genus Actinomyces Hp+/CagA- vs. Hp+/CagA+ 0.0107 0.0064 0.5352 0.5798

Genus Bacteroides Hp- vs. Hp+/CagA- 0.0236 0.0017 0.0184 0.0359 *

Genus Bacteroides Hp- vs. Hp+/CagA+ 0.0236 0.0024 0.0211 0.0374 *

Genus Bacteroides Hp+/CagA- vs. Hp+/CagA+ 0.0017 0.0024 0.4560 0.5231

Genus Fusobacterium Hp- vs. Hp+/CagA- 0.0281 0.0297 0.9525 0.9525

Genus Fusobacterium Hp- vs. Hp+/CagA+ 0.0281 0.0019 0.0256 0.0434 *

Genus Fusobacterium Hp+/CagA- vs. Hp+/CagA+ 0.0297 0.0019 0.2879 0.3490

Genus GemellaceaeOther Hp- vs. Hp+/CagA- 0.0333 0.0129 0.0711 0.1109

Genus GemellaceaeOther Hp- vs. Hp+/CagA+ 0.0333 0.0024 0.0058 0.0206 *

Genus GemellaceaeOther Hp+/CagA- vs. Hp+/CagA+ 0.0129 0.0024 0.1414 0.1902

Genus Granulicatella Hp- vs. Hp+/CagA- 0.0380 0.0073 0.0029 0.0140 *

Genus Granulicatella Hp- vs. Hp+/CagA+ 0.0380 0.0036 0.0015 0.0100 *

Genus Granulicatella Hp+/CagA- vs. Hp+/CagA+ 0.0073 0.0036 0.2266 0.2851

Genus Helicobacter Hp- vs. Hp+/CagA- 0.0052 0.7238 0.0000 0.0006 ***

Genus Helicobacter Hp- vs. Hp+/CagA+ 0.0052 0.9021 0.0000 0.0000 ***

Genus Helicobacter Hp+/CagA- vs. Hp+/CagA+ 0.7238 0.9021 0.0981 0.1471

Genus Neisseria Hp- vs. Hp+/CagA- 0.0294 0.0052 0.0210 0.0374 *

Genus Neisseria Hp- vs. Hp+/CagA+ 0.0294 0.0010 0.0097 0.0216 *

Genus Neisseria Hp+/CagA- vs. Hp+/CagA+ 0.0052 0.0010 0.1281 0.1785

Genus Porphyromonas Hp- vs. Hp+/CagA- 0.0240 0.0027 0.0080 0.0209 *

Genus Porphyromonas Hp- vs. Hp+/CagA+ 0.0240 0.0006 0.0049 0.0190 *

Genus Porphyromonas Hp+/CagA- vs. Hp+/CagA+ 0.0027 0.0006 0.0625 0.1016

Genus [Prevotella] Hp- vs. Hp+/CagA- 0.0349 0.0030 0.0078 0.0209 *

Genus [Prevotella] Hp- vs. Hp+/CagA+ 0.0349 0.0038 0.0089 0.0216 *

Genus [Prevotella] Hp+/CagA- vs. Hp+/CagA+ 0.0030 0.0038 0.6404 0.6750

Genus Prevotella Hp- vs. Hp+/CagA- 0.1855 0.0317 0.0113 0.0233 *

Genus Prevotella Hp- vs. Hp+/CagA+ 0.1855 0.0200 0.0077 0.0209 *

Genus Prevotella Hp+/CagA- vs. Hp+/CagA+ 0.0317 0.0200 0.4843 0.5396

Genus Rothia Hp- vs. Hp+/CagA- 0.0324 0.0178 0.2953 0.3490

Genus Rothia Hp- vs. Hp+/CagA+ 0.0324 0.0029 0.0073 0.0209 *

Genus Rothia Hp+/CagA- vs. Hp+/CagA+ 0.0178 0.0029 0.1994 0.2592

Genus Streptococcus Hp- vs. Hp+/CagA- 0.1896 0.0603 0.0100 0.0216 *

Genus Streptococcus Hp- vs. Hp+/CagA+ 0.1896 0.0185 0.0014 0.0100 *

Genus Streptococcus Hp+/CagA- vs. Hp+/CagA+ 0.0603 0.0185 0.1188 0.1716

Genus Veillonella Hp- vs. Hp+/CagA- 0.0619 0.0145 0.0015 0.0100 *

Genus Veillonella Hp- vs. Hp+/CagA+ 0.0619 0.0114 0.0006 0.0081 **

Genus Veillonella Hp+/CagA- vs. Hp+/CagA+ 0.0145 0.0114 0.7181 0.7370
