# Supplementary material for: The Human Gastric Microbiome Is Predicated upon Infection with Helicobacter pylori
Source: Front Microbiol. 2017 Dec 14;8:2508. doi: 10.3389/fmicb.2017.02508 (PMC5735373; doi:10.3389/fmicb.2017.02508)
Supplement: Supplementary file 3 [file Table3.DOCX]

**Supplementary Table 3:** Power calculations to determine the required sample size for statistically significant differences in three alpha diversity analysis calculation methods for the three gastric sample groups.

Type Comparison Effect SD N

chao1 H.p.- vs. H.p.+/CagA- 43.549 89.046 67

chao1 H.p.- vs. H.p.+/CagA+ 63.735 48.454 11

chao1 H.p.+/CagA- vs. H.p.+/CagA+ 20.186 89.046 307

observed_species H.p.- vs. H.p.+/CagA- 55.633 72.593 28

observed_species H.p.- vs. H.p.+/CagA+ 73.633 49.376 9

observed_species H.p.+/CagA- vs. H.p.+/CagA+ 18.000 72.593 257

PD_whole_tree H.p.- vs. H.p.+/CagA- 2.898 3.080 19

PD_whole_tree H.p.- vs. H.p.+/CagA+ 2.982 1.986 9

PD_whole_tree H.p.+/CagA- vs. H.p.+/CagA+ 0.084 3.080 21162
