# Supplementary material for: The Human Gastric Microbiome Is Predicated upon Infection with Helicobacter pylori
Source: Front Microbiol. 2017 Dec 14;8:2508. doi: 10.3389/fmicb.2017.02508 (PMC5735373; doi:10.3389/fmicb.2017.02508)
Supplement: Supplementary file 5 [file DataSheet1.docx]

Supplementary Material

THE HUMAN GASTRIC MICROBIOME IS PREDICATED UPON INFECTION WITH HELICOBACTER PYLORI

**Ingeborg Klymiuk^1^, Ceren Bilgilier^2^, Alexander Stadlmann^2^, Jakob Thannesberger^2^, Marie-Theres Kastner^2^, Christoph Högenauer^3^, Andreas Püspök^4^, Susanne Biowski-Frotz^5^, Christiane Schrutka-Kölbl^5^, Gerhard G. Thallinger^6,7^, Christoph Steininger^2*^**

*** Correspondence:** Corresponding Author: christoph.steininger@meduniwien.ac.at

# Supplementary Figures and Tables

## Supplementary Tables

**Supplementary Table 1**: Two-sided t-test and adj.pvalues of those taxa with significant differences in their relative abundances between the three sample groups from phylum to genus level. Significance: * adj.pvalue <0.05, ** adj.pvalue <0.01 and *** adj.pvalue <0.001.

**Supplementary Table 2**: List of taxa over all hierarchical levels found in microbiological analysis in the 29 samples used for analysis.

**Supplementary Table 3**: Power calculations to determine the required sample size for statistically significant differences in three alpha diversity analysis calculation methods for the three gastric sample groups.

**Supplementary Table 4**: Genera found to be unique for the three sample groups H.p.-, H.p.+/CagA- and H.p.+/CagA+, respectively.

## Supplementary Figures

**
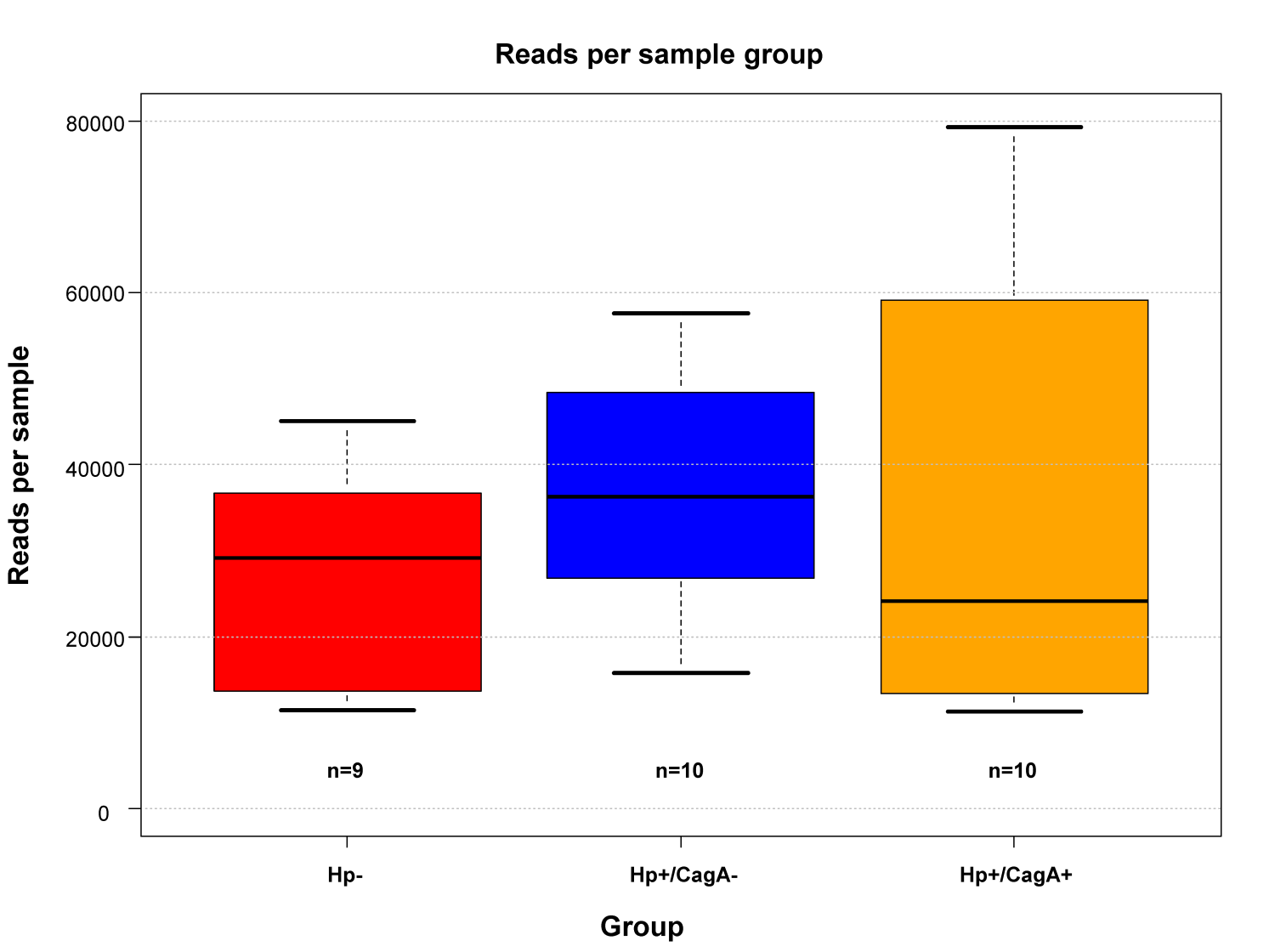
**

**Supplementary Figure 1.**

Box plot of the median reads distribution for each sample group after QC filtering, but before rarefaction to 11.200 reads per sample. The H.p.- group has a median number of reads of 29.111 (IQR=[13.651, 36.680]), H.p.+/CagA- a median of 36.255 (IQR=[26.728,48.502]), and H.p.+/CagA+ a median of 24.110 (IQR=[13.310,59.142]). P022-Z13 was an outlier in the H.p.+/CagA- group with 188.261 reads and therefore excluded from this figure.
